# The Novel Methylation Biomarker SCARA5 Sensitizes Cancer Cells to DNA Damage Chemotherapy Drugs in NSCLC

**DOI:** 10.3389/fonc.2021.666589

**Published:** 2021-06-04

**Authors:** Qi Peng, Yan Liu, Xuehua Kong, Jie Xian, Lin Ye, Li Yang, Shuliang Guo, Yan Zhang, Lan Zhou, Tingxiu Xiang

**Affiliations:** ^1^ Ministry of Education Key Laboratory of Diagnostic Medicine, and School of Laboratory Medicine, Chongqing Medical University, Chongqing, China; ^2^ Key Laboratory of Molecular Oncology and Epigenetics, The First Affiliated Hospital of Chongqing Medical University, Chongqing, China; ^3^ Department of Respiratory & Critical Care Medicine, The First Affiliated Hospital of Chongqing Medical University, Chongqing, China

**Keywords:** tumor marker, SCARA5, CpG methylation, FOXM1, non-small cell lung cancer

## Abstract

**Background:**

Scavenger Receptor Class A Member 5 (SCARA5), also known as TESR, is expressed in various tissues and organs and participates in host defense. Recent studies have found SCARA5 to produce an anti-tumor effect for multiple tumors, although the mechanistic basis for the effect is unknown.

**Methods:**

Bioinformatics, methylation-specific polymerase chain reaction (MSP), quantitative real-time PCR, and immunohistochemistry were used to assess promoter methylation and expression of SCARA5 in lung cancer tissues and cell lines. The biological effect of SCARA5 on lung cancer cells was confirmed by the CCK8 assay, colony formation assay, and flow cytometry. GSEA, Western blot, RNA sequencing, and luciferase-based gene reporter assay were used to explore the mechanistic basis for the anti-tumor effect of SCARA5. Chemosensitivity assays were used to evaluate the anti-tumor effect of SCARA5 in conjunction with chemotherapeutic drugs.

**Results:**

We found SCARA5 to be downregulated in lung cancer cell lines and tissues with SCARA5 levels negatively related to promoter methylation. Ectopic expression of SCARA5 suppressed proliferation of lung cancer both *in vitro* and *in vivo* through upregulation of HSPA5 expression, which inhibited FOXM1 expression resulting in G2/M arrest of the A549 cell line. SCARA5 also improved susceptibility of A549 cells to chemotherapeutic drugs that damage DNA.

**Conclusion:**

SCARA5 was silenced in NSCLC due to promoter methylation and could be a potential tumor marker in NSCLC.

## Introduction

Lung cancer is one of the most malignant tumors. Based on statistics released by the American Cancer Society’s Epidemiology Research Program in 2019, lung cancer incidence was ranked second among all malignant tumors with the mortality rate for lung cancer ranked first (1). Most early lung cancer patients choose surgical resection, which has 5-year survival rates ranging from 80% for stage IA to 50% for stage IIB (2). Unresectable advanced lung cancer patients receive a combination of radiotherapy and chemotherapy with a survival rate of 15% (3). Hence, intensive investigations are ongoing to mechanistically understand lung cancer development and as well to identify new and more effective treatments for the disease.

Scavenger receptors, a subclass of pattern recognition receptors (4), participates in pathogen clearance (5), lipid degradation (6), transmembrane transport (7), and a variety of other biological processes of the human body. At present, there are at least eight types of scavenger receptors (A–H) (8). SCARA5 is a class A scavenger receptor and encoded on chromosome 8 (9). SCARA5 expression is limited to testicular, airway, and thymic epithelial cells and is essential to host defense (10). SCARA5 has been shown to mediate iron delivery by transporting ferritin into renal epithelial cells (11). Recent studies have reported SCARA5 methylation levels to be elevated and expression levels to be decreased in liver cancer and breast cancer (12, 13). SCARA5 expression inhibits proliferation and migration of tumor cells and is considered a tumor suppressor gene (14–16). However, a role SCARA5 in lung cancer is not established.

FOXM1 (Forkhead Box M1) is a common transcription factor (17) that has four mRNA splice variant isoforms involved in mammalian cells proliferation (18). FOXM1a is inhibitory whereas FOXM1b and FOXM1c promote transcription (19). FOXM1d is located in the cytoplasm where it promotes colon cancer metastasis and invasion by accelerating cytoskeleton rearrangement and endothelial to mesenchymal transition (EMT) (20). Among the isoforms, FOXM1b is highly expressed in a variety of tumor cells (21–23) and functions as an oncogene, and regulates downstream target genes that determine multiple events within cancer cells (24, 25). Heat shock protein 70 (HSP70) binds FOXM1, which inhibits mRNA and protein expression, and as such acts as a cancer suppressor gene (26). Heat shock proteins are a class of molecules that regulate protein folding and prevent misfolding during stress. HSP70 is distributed within the nucleus and endoplasmic reticulum, where it maintains cell homeostasis (27–29) and protects against apoptosis (30). The role of HSP70 in tumor cells is complex and requires further definition.

In this study, for the first time, we found that methylation of the SCARA5 promoter silenced lung cancer gene expression. The ectopic expression of SCARA5 inhibited the proliferation of lung cancer cells *in vitro* and the growth of xenograft tumors *in vivo*. Further analysis showed that SCARA5 inhibits FOXM1 expression. RNA-sequence analysis demonstrated SCARA5 to upregulate HSP70 family member proteins and to enhance the sensitivity of A549 cell line to chemotherapy drugs. Thus, for non-small cell lung cancer (NSCLC), SCARA5 acts as a tumor suppressor that may serve as a marker for cancer prognosis and for clinical guidance during chemotherapy.

## Material and Methods

### Cells and Tissue Samples

All cell lines (A549, H1299) were obtained from the American Type Culture Collection (ATCC, Manassas, VA, USA) and supported with RPMI 1640 medium (Gibco-BRL, Karlsruhe, Germany) containing 1% penicillin and 10% fetal bovine serum (FBS) at 37°C/5% CO_2_, as recommended by ATCC. All tissue samples were obtained from the First Affiliated Hospital of Chongqing Medical University. This study was authorized by the Institutional Ethics Committees of the First Affiliated Hospital of Chongqing Medical University (Approval notice: # 2016-75) and abided by the Declaration of Helsinki.

### 5-Aza-2′-Deoxycytidine Treatment

Cell lines were treated with final concentration of 10 μM of 5-aza-2′-deoxycytidine (Aza, Sigma-Aldrich, Steinheim, Germany), which is a DNA methyltransferase inhibitor. After 3 days, RNA was collected for detection.

### MSP

Extracted the DNA of tissue and cell, then diluted the sample DNA (up to 1 μg) to 50 μl water in 1.5 ml microcentrifuge tube; added 5.5 μl 2 mol/L NaOH, 37°C, incubating for 10 min; add 10 mmol/L hydroquinone in 3 μ fresh configuration and 3 mol/L sodium bisulfite in 520 μl fresh configuration; mix and add enough mineral oil (about 50 μl) to cover the water phase, incubate at 50°C for 16 h. Remove the oil, add 1 ml DNA wizard reagent, and add the mixture to the miniprep column with the kit. Vacuum treatment, wash with 2 ml 80% isopropanol; add 55 ul 3 mol/L NaOH to each tube, incubate at room temperature for 5 min; add 1 μl 10 mg/ml glycoside with glycosyl group, and then add 17 μl 10 mol/L amine acetate and three volumes of 100% frozen ethanol. At 20°C, precipitate the DNA for several hours or overnight, centrifugate it for 20 mm, remove the supernatant, wash it with 70% frozen ethanol, add 20–30 μl of water to dissolve it; prepare the main reaction mixture of methylation and non-methylation PCR reaction (50 ul), take 3.8 ul of the main reaction mixture to the labeled PCR tube. Add 2 μl sodium bisulfite modified DNA template to each tube; dilute 1.25 μ Taq DNA polymerase to 10 μl sterile distilled water for each sample; add it into 40 ul mixture through oil layer, blow gently, and carry out PCR amplification. The MSP Primer of SCARA5 was shown in **Additional file 1: Table 1**.

### RNA Extraction, Reverse Transcription (RT)-PCR and Real-Time PCR

Total RNA in cell lines and tissues was extracted with TRIzol Reagent (Invitrogen, Carlsbad, CA, USA) following the standard manufacturer’s procedure, Reverse transcription PCR was performed on a total of 1 µg RNA into 20 µl cDNA with RT Reagent (Promega, Madison, WI, USA). Real time PCR was performed using 2× SYBR Green qPCR Master Mix (Bimake) following the Touch Down Protocol: 95°C for 3 min, followed by three cycles (95°C for 20 s, 60°C for 10 s, 3°C reduction per cycle) and 35 cycles (95°C for 20 s, 55°C for 10 s, 72°C for 1 s) by CFX manager v2.1 (BioRad). The *p* value of each group was calculated by t-test. All the primers used for Real-time PCR were listed in **Additional file 1: Table 1**.

### Preparation of Vector- and SCARA5-Expressing Stable Cell Lines

SCARA5-expressing and Vector (pReceiver-M35) plasmid were purchased from GeneCopoeia. Vector and SCARA5-containing plasmid (4 μg) were transfected with 5 μl Lipofectamine 2000 (Invitrogen, Carlsbad, USA) into H1299 and A549 cell lines incubating with RPMI 1640 medium without serum or penicillin for 6 h. After 48 h, G418 (Amresco, Solon, OH, USA) was used to screen cells. The screening process sustained 2 weeks. Ectopic expression of SCARA5 was verified with western blot and Real Time-PCR.

### Cell Proliferation and Colony Formation Assays

H1299 and A549 cells with vector or SCARA5 stably transfected were transplanted in 96-well plates and each well contained 2,000 cells. Cell vitality was measured at 0, 24, 48, and 72 h with Cell Counting Kit-8 (CCK-8; Beyotime, Shanghai, China) by microplate reader and absorbance was set on 450 nm. In addition, the colony formation assay (CFA) was applied to measure cell proliferation. Vector and SCARA5-expressing cells were cultured in six-well plates at three densities (100, 200, and 400 cells/well) for 10 days. The cells were fixed with 4% paraformaldehyde for 15 min, washed with PBS for 5 min, and stained with crystal violet for 15 min.

### Flow Cytometry (FCM)

Vector and SCARA5-expressing Cells were fixed with 70% alcohol overnight before evaluating cell cycle. All the cells were stained with propidium iodide (PI) for 30 min. To assess apoptosis,we applied PI. A CellQuest kit (BD Biosciences, CA, USA) and annexin V-flurescein isothiocyanate to stain cells. Cell cycle and apoptosis were analyzed with flow cytometry (FCM). All experiments were repeated for three times.

### Chemosensitivity Assay

The effect of SCARA5 on the cytotoxicity of Gemcitabine, 5-fluorouracil (5-FU), and cisplatin was assayed using CCK-8. Briefly, SCARA5-expressing and vector stable A549 cells were plated at 4,000/well in 96-well plates. After 4 h, removed previous medium and added containing different concentrations of drugs. Cell vitality was assessed after 48 or 72 h with CCK-8 (Dojindo, Shanghai, China) following the standard procedures. Absorbance was set on 450 nm with a microplate reader. The half inhibitory concentration (IC50) of each drug was calculated with GraphPad Prism7.0. All experiments were repeated for three times.

### Luciferase-Based Gene Reporter Assay

pGL3-CCNB1, pGL3-CHK1, pGL3-CDC25C, and pcDNA3.1-FOXM1 were used for the reporter assays. Renilla plasmid as control. After transfecting for 48 h, Added the lysis buffer (100 µl/well), shaken vigorously for 15 min, and collected the cell lysate. Then 20 µl sample was detected with Dual-luciferase reporter assay kit (Promega, Madison, WI, USA). After adding 50 µl start, shake it slightly and test immediately, following with 50 µl stop solution and evaluate it again. The fluorescence value was quantified by Infinite M200 PRO luminometer (Tecan, Austria).

### Western Blot

We applied 10 and 12% concentration of sodium dodecyl sulfate polyacrylamide gel electrophoresis (SDS-PAGE) to separate protein lysates and transferred them onto polyvinylidene difluoride (PVDF) membranes (Bio-Rad, Hercules, CA, USA). Then, membranes were incubated with primary antibodies specific for SCARA5 (#ab118894, Abcam), FOXM1 (#ab207298), CyclinB1 (Sc245), CHK1 (A5004, Bimake), CDC25C (A5133, Bimake), CDC25C (Ser216) (#4901, CST), CDK1 (SC54), Phospho-HistoneH2AX (Ser139) (#80312, CST), HSPA5 (#3177, CST). Protein bands were exposured with Western Chemiluminescent HRP Substrate kit (Millipore Corporation, Billerica, MA, USA).

### Tumor Xenograft Model in Nude Mice

Six 4-weeks old Immunocompromised female nude mice were purchased from The Animal Center of Chongqing Medical University and using for xenograft studies. Stable SCARA5-expressing or vector A549 was digested to obtain single cell suspension. Each mouse was subcutaneously injected with 100 μl PBS containing 5 million cells. The tumor size was measured every two days after 7 days of injection. The raising condition was supported with national standards (Laboratory Animal-Requirements of Environment and Housing Facilities; GB14925-2010). The care and operation of experimental animals were in accordance with the Chongqing Management Approach of Laboratory Animals (Chongqing government order No. 195). All the mice were sacrificed after 19 days. Tumor volume (mm^3^) was calculated as follows: volume = length × width^2^ × 0.52. All the transplanted tumor was placed in 4% paraformaldehyde and then embedded with paraffin.

### Immunohistochemistry Staining

Expression of SCARA5 in paraffin embedded lung cancer, adjacent paracancerous tissues, and xenograft tumor tissues in mice was detected by immunohistochemistry staining. The tissue was cut into 4 μm thick slices, transferred onto glass, and incubated at 65°C for at least 6 h. All the required reagents except antibody came from an Immunohistochemistry Kit (ZSGB-BIO, Beijing, China) and operation was performed according to the standard procedures. Slides were incubated at 4°C for 16–20 h with Ki67 (#16667, Abcam) and SCARA5 (#ab118894, Abcam) antibodies. Then tissues were stained with DAB substrate (K176810E, ZSGB-BIO, China) for 40–50 s. The nuclei were stained with hematoxylin for 5 s and covered with neutral resin. The image of IHC was observed by microscope. IHC scoring criteria: 0: The positive rate was less than 10%; 1: Positive rate was between 10 and 30%; 2: Positive rate was between 30 and 50%; 3: Positive rate was more than 50% added a description of **Additional file 2: Table 2**.

### ER-Tracker and Immunofluorescence Staining

ER-Tracker Red kit (C1042S) was purchased from Beyotime. Cells were cultured in 24-well plate containing glass coverslips. After 48 h of transfection of SCARA5 plasmid, diluted ER tracker was added and incubated for half an hour in 37°C. Washed cells for 5 min, and fixed with 4% paraformaldehyde for 30 min, permeabilized for 5 min in 0.5% Triton X-100,and blocked with 5% BSA for 1 h at 25°C. Samples were incubated overnight with primary antibody of SCARA5 (#ab118894, abcam) at 4°C. The next day, washed the cells with PBS for three times (5 min once) and incubated with Alexa 488-conjugated goat anti-rabbit secondary antibody for 1 h (Avoid light). Nuclei were stained with DAPI (Roche, Palo Alto, CA, USA). Picked out the coverslip, added 4 μl anti-quenching agent, and covered the slide onto a glass observed with Laser scanning confocal microscope.

### RNA Sequencing

Vector and SCARA5-expressing A549 cells were cultured in 6 cm dishes. When the cell density reached 80%,RNA was collected and sent to the company for sequencing. The Novogene Co. Ltd company performed the actual sequencing work. The initial RNA was total RNA, and the total amount was more than 1 μg. Illumina NEBNext^®^ UltraTM RNA Library Prep Kit was used in the construction of the library. After the construction of the library, Qubit2.0 fluorometer and Agilent 2100 Bioanalyzer were used to detect the library to ensure the quality of the library. The image data measured by high-throughput sequencer was converted into reads by Casava base recognition, and the file was saved in fastq format. Then the annotation files of reference genome and gene model was downloaded from genome website, constructing the index of reference genome using hisat2 v2.0.5, and selected hisat2 as the comparison tool.

### Differential Expression Analysis

The expression differences between the two groups were analyzed by using deseq2r software (1.16.1). Benjamin and Hochberg were used to adjust the p value to control the error detection rate. Genes with adjusted P < 0.05 were assigned as differentially expressed genes by deseq2 (edge was used for those without biological duplication). Before differential gene expression analysis, for each sequencing library, the read count was adjusted by edge package through a scale normalization factor. Edge software package (3.18.1) was used for differential expression analysis of the two conditions. Benjamin & Hochberg method was used to adjust p value. The corrected p value and |log2foldchange | were used as the threshold of significant differential expression.

### Enrichment Analysis of Differential Genes

Go enrichment analysis of differentially expressed genes was realized by clusterProfiler R software, in which the gene length deviation was corrected. GO terms with a corrected p value of less than 0.05 were considered to be significantly enriched by differentially expressed genes. We used clusterProfiler R software to analyze the statistical enrichment of differentially expressed genes in KEGG pathway.

### Bioinformatics

GSEA was performed with GSEA software 4.0.3 as previously described (31). The gene expression dataset GSE12667 was downloaded from the GEO database (https://www.ncbi.nlm.nih.gov/geo/), which contained 71 LC cases. Samples were divided into two groups based on the median SCARA5 (229839_at) in lung cancer through Oncomine database (https://www.oncomine.org/resource/login.html). The SCARA5-high group contained 27 samples while SCARA5-low groups contained 47 samples. The gene set “c2.cp.reactome.v7.2.symbols” was used for the enrichment analysis. The number of permutations per gene set was set at 1,000 to obtain the normalized enrichment score (NES). A normal p-value <0.05 with false discovery rate <0.25 were considered significantly enriched. DEG of GSE12667 was used GEO2R. Venn diagram is made by using jvenn web (http://jvenn.toulouse.inra.fr/app/example.html).

### Statistical Analyses

Statistical analyses were performed with GraphPad Prism 7.0 and SPSS (version 22.0, SPSS, Chicago, IL, USA). Student test and Mann-Whitney U test were used to define p value. The data were considered significant when *p* < 0.05.

## Results

### SCARA5 Expression Is Downregulated in Lung Cancer and Related to Prognosis

**Figure 1:**
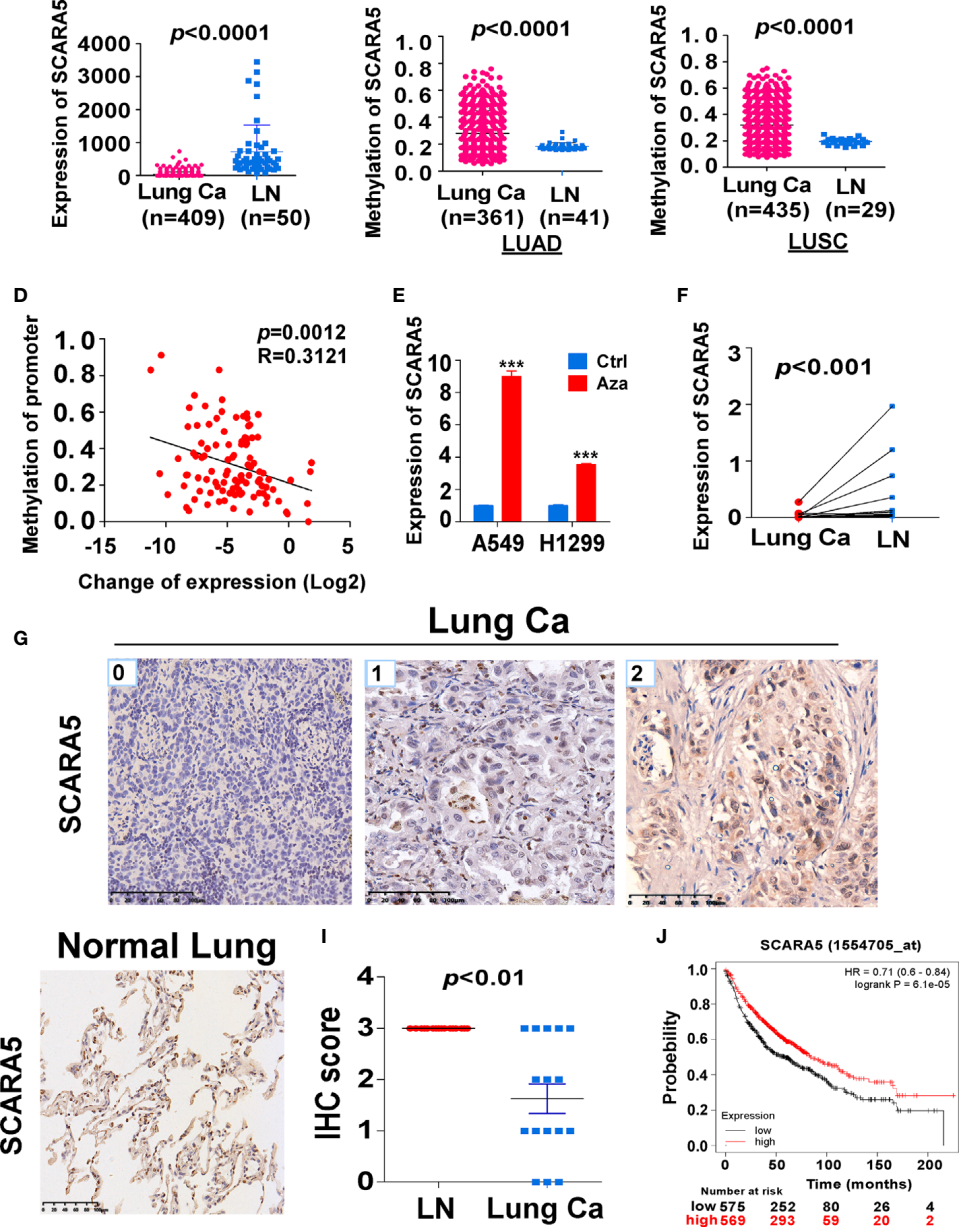
SCARA5 expression was downregulated in lung cancer and related to prognosis. **(A)** SCARA5 mRNA expression in lung cancer and paired non-tumor tissues from TCGA database (p < 0.0001). **(B, C)** Methylation of SCARA5 promoter in Lung adenocarcinoma (Lung squamous cell carcinoma) and paired paracancerous tissues in MethHC database (p < 0.0001). **(D)** Relation between methylation of promoter and fold change of SCARA5 expression (R = 0.3121, p = 0.0012). **(E)** SCARA5 Expression of lung cancer cells after treating with 5-aza for 72 h, (***p < 0.001). **(F)** mRNA expression of SCARA5 in 11 cases of clinical lung cancer and paired non-tumor tissues (p < 0.001). **(G)** Immunohistochemical staining of SCARA5 in cancer tissue, Left was cancer tissue with a IHC score of 0; Middle score was 1; Right score was 2. **(H)** Immunohistochemical staining of SCARA5 in paracancerous tissue was positive with a score of 3. **(I)** Statistical chart of SCARA5 immunohistochemical score of tumor and paracancerous tissues (p < 0.01). **(J)** Overall survival of lung cancer patients with high or low expression of SCARA5 from KM plotter database (p < 0.001).

In order to understand the role of SCARA5 in lung cancer, we conducted a series of bioinformatics analyses. First, we analyzed SCARA5 expression in 409 lung cancer tissues (including 50 paired normal lung tissues) using The Cancer Genome Atlas (TCGA) database. SCARA5 expression was significantly downregulated in tumor tissues compared to normal lung tissues within the MethHC database (*p* < 0.001; **Figure 1A**). Since promoter methylation is one of the common mechanisms for gene silencing, we analyzed the methylation of the SCARA5 promoter in 435 lung squamous cell carcinoma tissues (including 29 normal lung tissues) and 361 lung adenocarcinoma tissues (including 41 normal lung tissues). Hypermethylation of the SCARA5 promoter in these types of lung cancer was significantly related to decreased expression of SCARA5 (**Figures 1B–D**). SCARA5 gene expression was low in A549 and H1299 cell lines and was upregulated after treatment with 5-Aza-2’-deoxycytidine (**Figure 1E**). Kaplan-Meier survival curve analysis demonstrated that patients with low levels of SCARA5 expression had poorer overall survival than patients with high levels of SCARA5 expression (**Figure 1J**).

**Figure 2:**
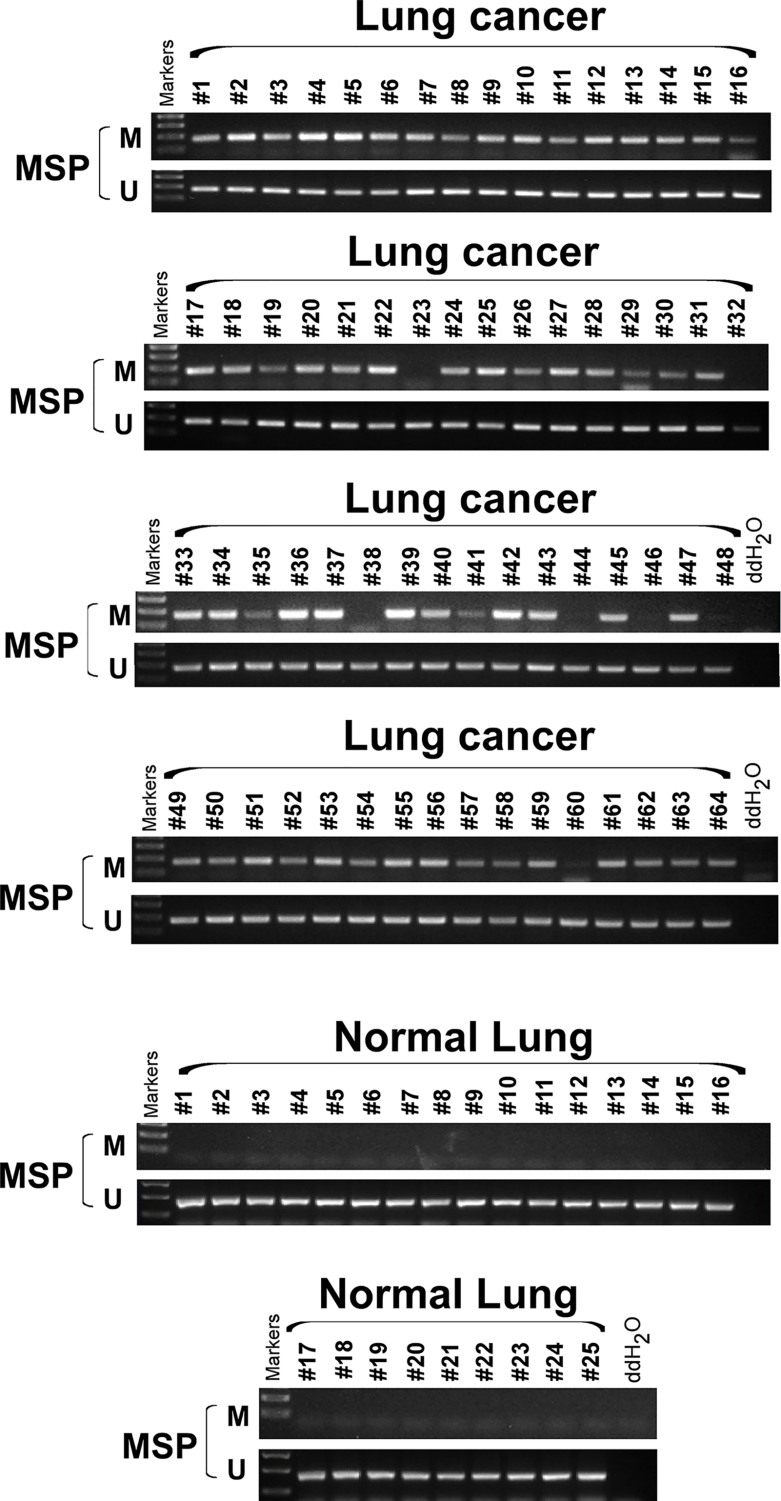
SCARA5 promoter was hypermethylated in lung cancer. Methylation status of 64 lung cancer tissue and 25 normal tissue sample was detected by Methylation specific PCR (MSP), 57/64 (89%) primary tumor tissues detected hyper- methylation of promoter, but none of normal lung tissues was detected hypermethylation.

We extracted mRNA from 12 paired clinical lung cancer and paracancerous tissues. Quantitative reverse transcription polymerase chain reaction (RT-PCR) revealed that SCARA5 expression in tumor tissue was significantly lower than in adjacent non-tumor tissue (**Figure 1F**). SCARA5 protein was also detected by immunohistochemistry (IHC). Compared to normal tissue, SCARA5 immuno-reactivity was significantly lower in lung cancer (**Figures 1G–I**). SCARA5 methylation status of 64 lung tumor tissues and 16 normal tissues was assessed by methylation specific PCR (MSP). Promoter hypermethylation was found in 57/64 (89%) of primary tumor tissues and 0/25 normal lung tissues, which is consistent with the database (**Figure 2**). Collectively, these data suggest that SCARA5 is downregulated in lung cancer due to promoter methylation and that SCARA5 downregulation is positively related to prognosis.

### SCARA5 Suppresses Lung Cancer Growth *In Vitro* and *In Vivo*

**Figure 3:**
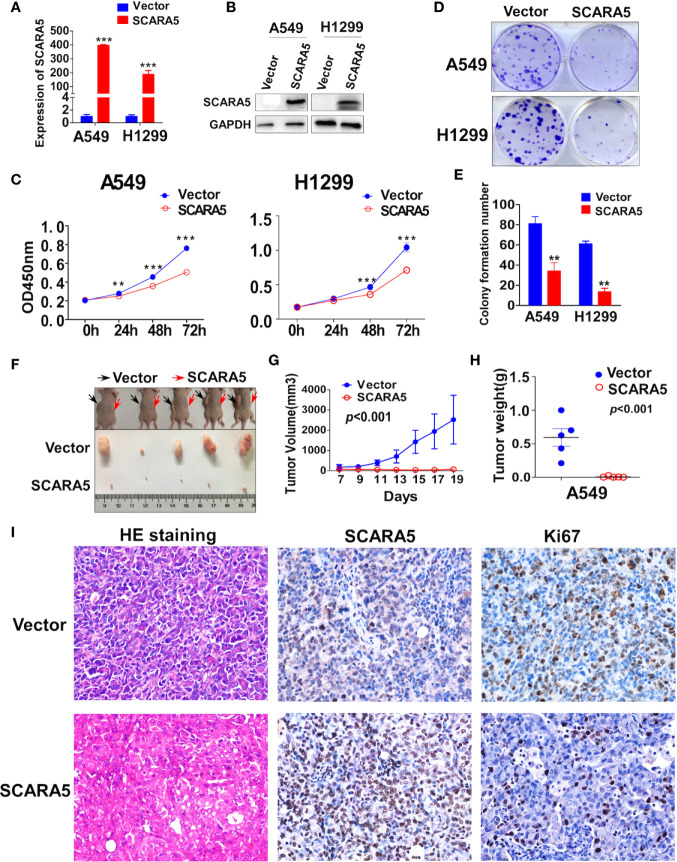
SCARA5 suppressed lung cancer cell proliferation *in vitro* and *in vivo*. **(A, B)** Overexpression of SCARA5 in A549 and H1299 were confirmed by quantitative PCR and western blot. **(C)** Inhibitory effect of SCARA5 on A549 and H1299 was measured by CCK8. **(D, E)** Images and statistical chart of the colony formation assay in vector and SCARA5-expressed A549 and H1299 cells. Data was presented as mean ± SD, **p < 0.01, ***p < 0.001. **(F, G)** Vector and SCARA5-expressed A549 were Subcutaneously injected into 4-week female nude mice. Tumor size was measured every 2 days. All the mice were sacrificed at 19 days. **(H)** The tumor weight was measured (p < 0.001). **(I)** HE staining, Immunohistochemical staining of SCARA5 and ki67 in vector and SCARA5-expressed xenografts was presented, respectively.

To investigate the function of SCARA5 in lung cancer, H1299 (p53 null) and A549 cells (p53 wild type) were transfected with a plasmid that overexpressed SCARA5. Control was simply transfection with vector. The efficiency of overexpression was verified by RT-PCR and western blot (**Figures 3A, B**). Results demonstrated SCARA5 to significantly inhibit the proliferation of both A549 and H1299 cells (**Figures 3C–E**).

The inhibitory effect of SCARA5 on lung cancer *in vivo* was assessed. Cells over expressing SCARA5 or control A549 cells were injected subcutaneously into nude mice. Compared to the control group, xenografts of cells over expressing SCARA5 had smaller mean volumes and reduced tumor weights (**Figures 3F–H**). SCARA5 overexpression in the xenografts was assessed by IHC. Ki67 staining of tumors demonstrated significantly reduced proliferative activity. Hematoxylin and eosin (HE) staining demonstrated a significantly increased quantity of smaller cells with darker, more irregular nuclei (**Figure 3I**). Taken together, these data suggest that SCARA5 has an Inhibitory effect on lung cancer cells both *in vitro* and *in vivo.*

### SCARA5 Induced Cell Cycle Arrest and Apoptosis in Lung Cancer Cells

**Figure 4:**
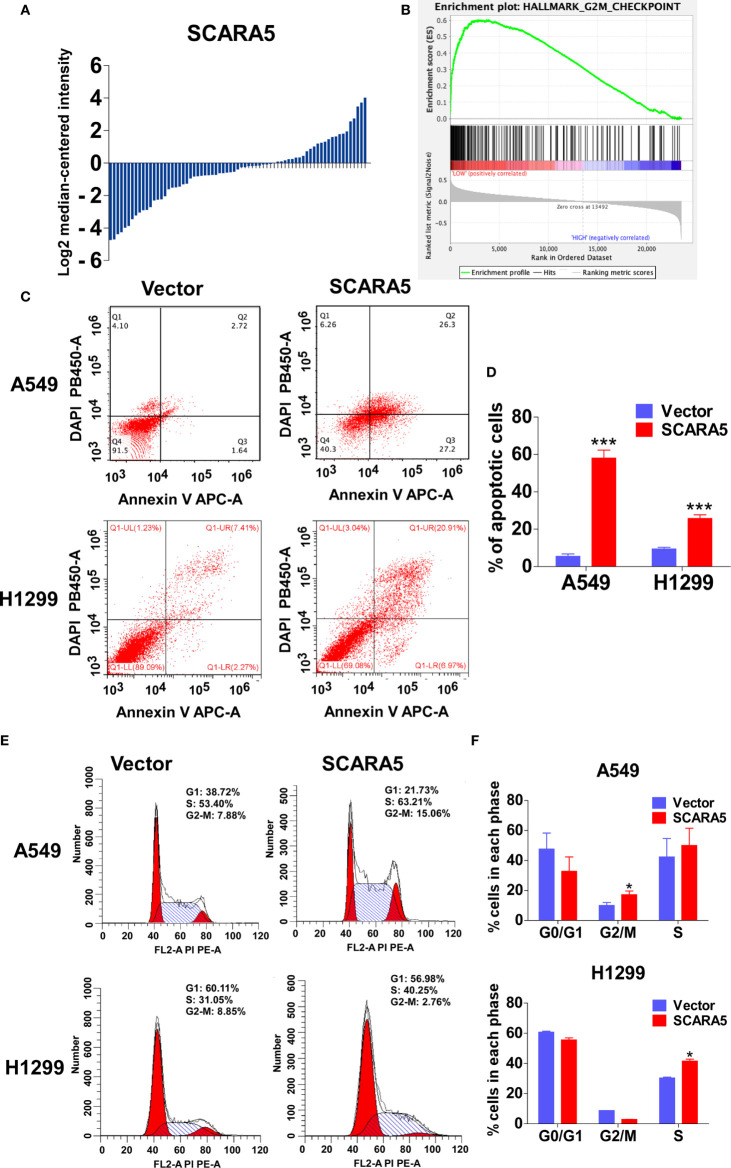
SCARA5 induced cell cycle arrest and apoptosis in lung cancer. **(A)** The SCARA5 expression of 71 lung cancer samples in GSEA12667 was arranged by log2 median intensity. **(B)** Gene enrichment plots showed that the gene set G2M_CHECKPOINT was enriched in SCARA5-Low subgroup. **(C, D)** The effects of SCARA5 on apoptosis in A549 and H1299 cells were detected by flow cytometry analysis too. Representative flow cytometry plots and histogram statistics of apoptosis changes. **(E, F)** The effects of SCARA5 on cell cycle in A549 and H1299 cells were detected by flow cytometry analysis. Representative flow cytometry plots and histogram statistics. Data was presented as mean ± SD, *p < 0.05, ***p < 0.001.

In order to explore the mechanistic basis for the effect of SCARA5 on lung cancer, we divided the samples of GEO datasets GSE12667 and GSE2109 into two categories according to the median expression of SCARA5 (**Figure 4A**), and Gene Set Enrichment Analysis (GSEA) was performed with the GEO datasets GSE12667 and GSE2109. Results with both datasets demonstrated SCARA5 function to be related to G2/M cell cycle arrest (**Figure 4B**). Flow cytometry showed SCARA5 to promote apoptosis of A549 and H1299 cells when compared to control (**Figures 4C, D**). The proportion of cells in the G2/M phase was increased in A549 cells overexpressing SCARA5 when compared to vector control. However, the same phenomenon was not observed for H1299 cells, wherein SCARA5 induced S phase cell cycle arrest (**Figures 4E, F**). These data suggest that SCARA5 can inhibit cell proliferation by inducing apoptosis and by blocking cell cycle progression in lung cancer cell lines.

### SCARA5 Induces G2/M Cell Cycle Arrest in A549 Cells by Inhibition of FOXM1

**Figure 5:**
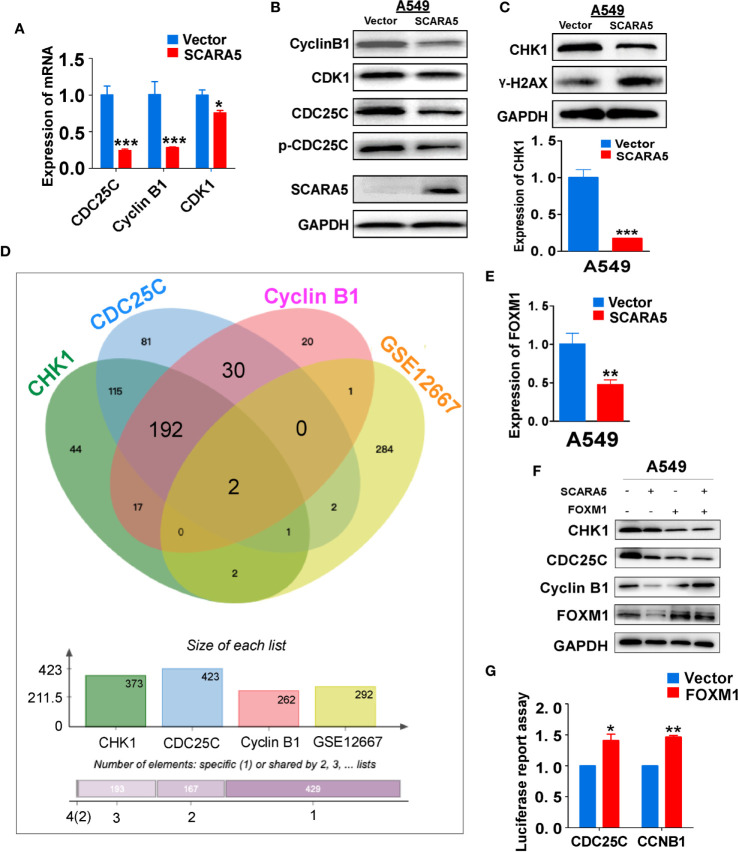
SCARA5 induced G2/M cell cycle arrest by inhibiting FOXM1. **(A, B)** The inhibitory effect of SCARA5 on cycle related markers was verified by q-PCRand WB. **(C)** After overexpression of SCARA5, the DNA damage repair marker phosphorylated histone H2AX and checkpoint protein was detected by q-PCR and WB. **(D)** The common transcription factors of CyclinB1, CDC25C, CHK1, and DEGs of GSE12667 was shown in Venn diagram. **(E)** The mRNA expression of FOXM1 was downregulated in SCARA5-expressed A549 cells. **(F)** Ectopic expression of FOXM1 attenuated the inhibitory effect of SCARA5 on CDC25C, Cyclin B1, and CHK1. **(G)** FOXM1 protein could combine with the promoters of CyclinB1 and CDC25C. *p < 0.05, **p < 0.01, ***p < 0.001.

CDK1 functions as an important kinase during the G2 phase (32). It is known that CDK1 becomes activated after binding to CyclinA2 or CyclinB1 (33). The activity of CDK1 is regulated by three phosphorylation sites; Thr14, Tyr15, and Thr16. Thr14 and Tyr15 inhibit the activity of CDK1, which requires dephosphorylation during the G2/M phase (34). CDC25C promotes the dephosphorylation of Thr14 and Tyr15, thus enabling CDK1 to become active and to promote G2/M cell cycle progression. Whereas, Wee1 and MyT1 inhibit dephosphorylation and induce G2/M arrest (35). CDC25C is phosphorylated and inactivated by checkpoint kinase1 (CHK1, also known as CHEK1) (36). To investigate the mechanism of G2/M cell cycle arrest induced by SCARA5, we examined the mRNA and protein levels of CDK1, CDC25C, and CyclinB1 in the SCARA5 overexpressing cell line. We found that mRNA and protein levels of these three indicators were downregulated (**Figures 5A, B**). Furthermore, we assessed the expression of CHK1 and found that both mRNA and protein levels were significantly down regulated when the DNA damage marker γ-H2AX (37) was increased (**Figure 5C**). To understand the downregulation of CHK1, we analyzed the common transcription factors for CDC25C, CHK1, and CyclinB1. These were compared to the differential genes of GSE12667 and in this manner two genes FOXM1 and FOS1 were identified (**Figure 5D**). FOXM1 was downregulated in SCARA5-overexpressing A549 cells (**Figure 5E**), but no change in FOS1 expression was detected (data are not shown). A luciferase reporter assay confirmed binding of FOXM1 to the promoters of CDC25C and CyclinB1 (**Figure 5G**). Next, we transfected FOXM1 plasmid into SCARA5 overexpressing A549 cells and the inhibitory effect of SCARA5 on CDC25C, Cyclin B1, and CHK1 was alleviated (**Figure 5F**). Taken together these data demonstrate SCARA5 to inhibit FOXM1, a transcription factor for CDC25C and CyclinB1, resulting in G2/M cell cycle arrest of A549 cells.

### SCARA5 Is Associated With Endoplasmic Reticulum Function and the Upregulation of HSP70 Family Member Proteins

**Figure 6:**
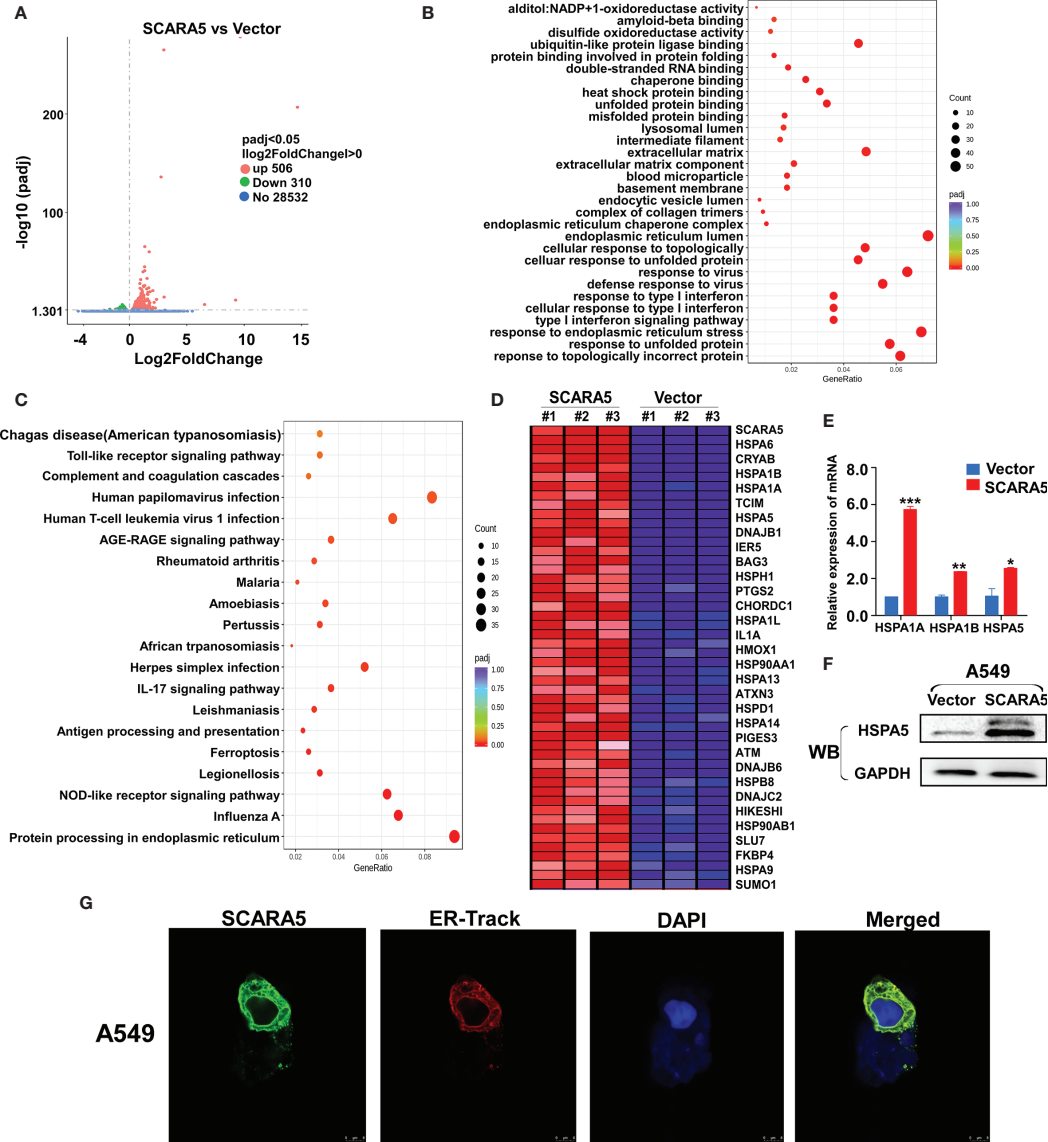
SCARA5 was associated with endoplasmic reticulum function and induced HSP70 family proteins upregulated. **(A)** Volcano map of DEGs in vector- and SCARA5-expressed A549 cells. **(B, C)** Go and KEGG analysis showed SCARA5 related to endoplasmic reticulum and unfold protein reaction. **(D)** DEGs related to UPR were mainly Heat shock protein family proteins. **(E, F)** Upregulation of HSP70 in SCARA5-expressed A549 cells was confirmed by q-PCR and WB. **(G)** Confocal microscopy showed subcellular localization of SCARA5 was similar to ER. *p < 0.05, **p < 0.01, ***p < 0.001.

The means by which SCARA5 regulates FOXM1 was explored by RNA sequencing of SCARA5- and vector-transfected A549 cells. As shown by volcano maps and thermograms, 506 genes were upregulated and 310 genes were downregulated in A549 cells after overexpression of SCARA5 (**Figure 6A**). Go analysis showed that differential expressed genes (DEGs) were related to the unfolded protein reaction, while KEGG showed that the DEGs were mainly related to endoplasmic reticulum protein function (**Figures 6B, C**). The DEGs related to UPR were mainly HSP70 protein family members including; HSPA1A, HSPA1B, HSPA5, and HSPA6 (**Figure 6D**). mRNA and protein levels for HSP70 and HSPA5 were verified by qRT-PCR and western blot analysis, respectively (**Figures 6E, F**). Furthermore, we found that SCARA5 protein was localized in the endoplasmic reticulum (**Figure 6G**). These data indicate that SCARA5 is associated with UPR and induces HSP70.

### In A549 Cells, SCARA5 Induces Sensitivity to Drugs That Damage DNA

**Figure 7:**
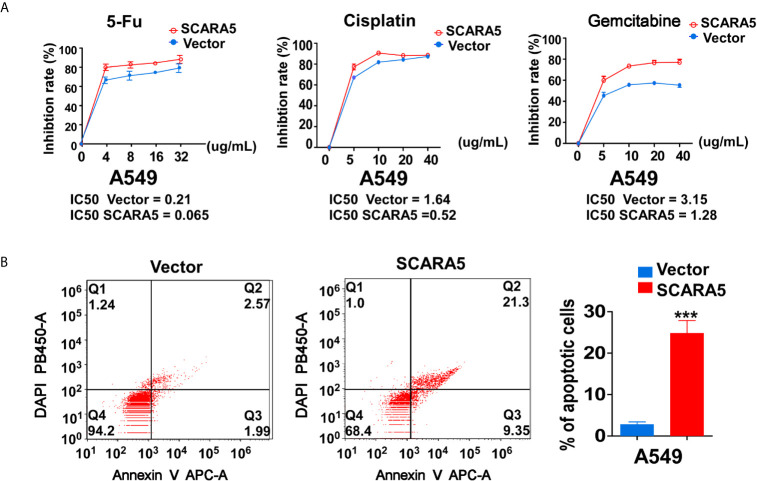
SCARA5 induced sensitivity of A549 to DNA damage drugs. **(A)** Inhibition curves and IC50 of A549 cells under different concentrations of 5FU (Left), Cisplatin (Middle), Gemcitabine (Right). **(B)** After treating 48 h with 5FU, apoptotic cells were detected by flow cytometry analysis. Representative flow cytometry plots (Left) and histogram statistics (Right). ***p < 0.001.

In that CHK1 is the essential gene for DNA damage repair during cell cycle progression, CHK1 inhibitors have been developed. Many investigations have demonstrated CHK1 inhibitors to effectively enhance the sensitivity of various cancer cells to chemotherapeutic drugs (38, 39). In this study, we found that SCARA5 inhibited CHK1 expression, so three types of chemotherapeutic drugs that induce DNA damage were used to treat A549 cells overexpressing-SCARA5. As expected, SCARA5 significantly increased the chemosensitivity of tumor cells to 5-fluorouracil, Cisplatin, and Gemcitabine (**Figure 7A**). After treatment with 5-fluorouracil for 48 h and as judged by flow cytometry, a greater proportion of apoptosis was found in SCARA5 overexpressing A549 cells than in vector-A549 cells (**Figure 7B**). The same phenomenon was observed in A549 cells treated with Cisplatin and Gemcitabine (data are not shown). These data suggest that SCAR5 enhances sensitivity of A549 cell lines to chemotherapeutic drugs that cause DNA damage.

## Discussion

In recent years, liquid biopsy has become more and more common place. As a part of liquid biopsy, detection of DNA methylation plays an important role in guiding clinical treatment. In breast and liver cancer, promoter hypermethylation of SCARA5 results in low gene expression (12, 13), which promotes the process of tumor malignancy. By use of the TCGA database, we found SCARA5 expression to be low in lung cancer and high in paracancerous tissues and this observation was related to hypermethylation of the SCARA5 promoter (**Figures 1A, B**). By detection of methylation and gene expression levels in lung cancer tissues, we found the promoter of SCARA5 to be hypermethylated (**Figure 2**) and SCARA5 protein levels to be decreased (**Figure 1I**), which is consistent with the database results. After demethylation, the expression of SCARA5 in lung cancer cells was upregulated (**Figure 1E**), which indicates that SCARA5 hypermethylation results in low SCARA5 gene expression. Taken together these data suggest that promoter methylation of the SCARA5 gene results in SCARA5 gene silencing in lung cancer.

**Figure 8:**
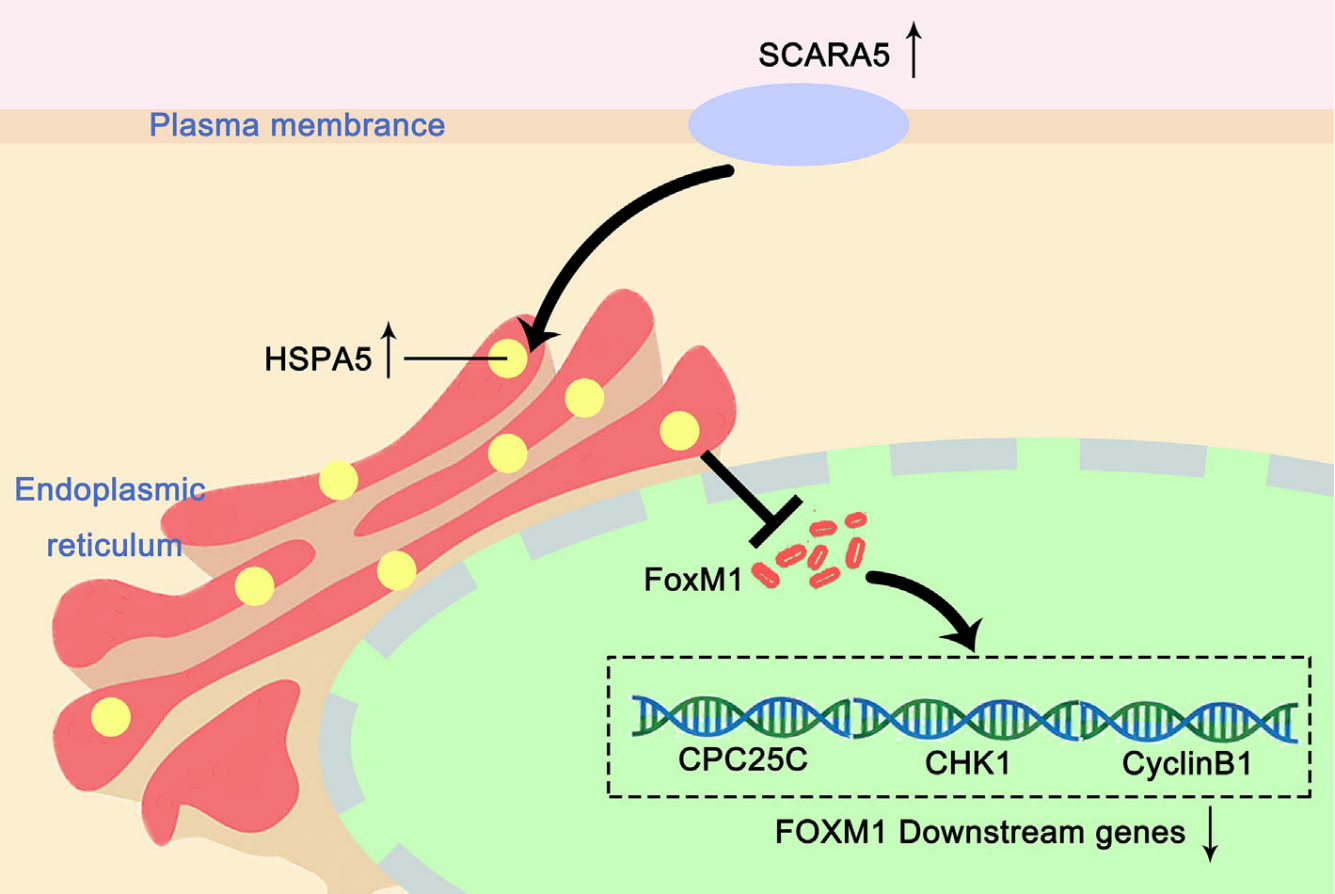
Proposed mechanism of how SCARA5 affects HSP70/FOXM1/CCNB1 in A549. SCARA5 is expressed on the cell membrane and enters into the cytoplasm through endocytosis and locates in the endoplasmic reticulum, causing UPR, leading to the upregulation of HSP70 family protein. HSP70 inhibits the expression of FOXM1, which leads to the downregulation of CyclinB1 and CDC25C, the downstream genes of FOXM1, causing G2/M arrest and inhibits the proliferation of lung cancer cells.

In this study, we found that re-expression of SCARA5 can inhibit cell proliferation, reduce lung cancer xenograft tumor growth, and arrest A549 cells in the G2/M phase of the cell cycle. As well, bioinformatics analysis found SCARA5 to be related to G2/M cell cycle arrest. However, in H1299 cells SCARA5 induced S phase arrest, which was unlike results with A549 cells. In general, when DNA is damaged, the ATM-CHK1 signal pathway is activated and CHK1 phosphorylates and inactivates CDC25C. Thus, phosphorylated CDC25C cannot dephosphorylate Thr14 and Tyr15 of CDK1, which are the inhibitory phosphorylation sites, resulting in cell cycle arrest in G2/M. SCARA5 was found to inhibit expression of CyclinB1 and CDC25c, and as well to induce G2/M cell cycle arrest and also unexpectedly to suppress CHK1. FOXM (a common transcription factor for CyclinB1, CDC25C, and CHK1) was found to be differentially expressed in the GEO dataset GSE12667. R sequence showed SCARA5 to induce HSP70 family member proteins, and to be distributed in the endoplasmic reticulum (HSPA5, known as GRB78) and in the nucleus (HSP70). These proteins participate in the unfolded protein reaction during stress conditions and are considered anti-apoptotic in cancer cells. SCARA5 induced apoptosis in A549 and H1299 cells, leading to upregulation of HSP70. It has been reported that HSP70 binds to FOXM1, suppressing its transcription (26). Since HSP70 is the target gene of TP53 (40) and since TP53 is absent in the H1299 cell line, we speculate that this absence may be the reason for inconsistent results between the two cell lines. We transferred TP53 and SCARA5 plasmids into H1299 cells. SCARA5 inhibited FOXM1 expression in the presence of p53 in H1299 cells. However, SCARA5 had no effect on G2/M cycle marker proteins with or without TP53 in H1299 cells. Thus, we conclude that SCARA5 induces apoptosis and cell cycle arrest in lung cancer cells. SCARA5 upregulated HSP70 family member proteins, which inhibited transcription factor FOXM1 expression, resulting in G2/M cell cycle arrest in A549 cells (**Figure 8**). Cisplatin, Gemcitabine, and 5-fluorouracil are first-line chemotherapy drugs for lung cancer. However, drug resistance limits their curative effect (41, 42). SCARA5 overexpression increased sensitivity of A549 to these drugs by downregulation of CHK1, which is reported to be related to chemotherapy resistance.

In summary, SCARA5 was silenced in non-small cell lung cancer by promoter methylation, which related to prognosis, apoptosis, and cell cycle arrest in lung cancer cells. Although we only clarified the mechanism in A549 cell line, these results suggest that SCARA5 is a cancer suppressive factor which may be a potential tumor marker for NSCLC. By analysis of the methylation levels of SCARA5, we may be better able to evaluate patient prognosis and as well provide improved clinical chemotherapy.

## Data Availability Statement

The raw data supporting the conclusions of this article will be made available by the authors, without undue reservation.

## Ethics Statement

The studies involving human participants were reviewed and approved by the Institutional Ethics Committee of the First Affiliated Hospital of Chongqing Medical University (#2016-61). The patients/participants provided their written informed consent to participate in this study. The animal study was reviewed and approved by Institutional Ethics Committee of the First Affiliated Hospital of Chongqing Medical University.

## Author Contributions

LZ and TX: conception and design. QP, YL, and XK: performed majority of experiments. JX and TX: performed experiments and analyzed data. LYe and LYa: collected samples. QP, LZ, and TX: drafted the manuscript. LZ, TX, and SG: reviewed data and finalized the manuscript. All authors contributed to the article and approved the submitted version.

## Funding 

This study was supported by National Natural Science Foundation of China (#81872380, #81572769), Natural Science Foundation of Chongqing (CYB19161), and open fund of Key Laboratory of Molecular Oncology and Epigenetics (2019-07).

## Conflict of Interest

The authors declare that the research was conducted in the absence of any commercial or financial relationships that could be construed as a potential conflict of interest.

## Abbreviations

LUAD, Lung adenocarcinoma; LUSC, Squamous cell carcinoma of lung; 5-FU, 5-Fluorouracil; CCK8, Cell Counting Kit-8; FOXM1, Forkhead Box M1; Aza, 5-Aza-2-deoxycytidine; SCARA5, Scavenger Receptor Class A Member 5; CDC25C,Cell division control protein 25C; CHK1, Checkpoint kinase1; RT-PCR, Reverse transcription Polymerase Chain Reaction; PVDF, Polyvinylidene fluoride; IC50, half maximal inhibitory concentration; SDS-PAGE, Sodium dodecyl sulfate-polyacrylamide gel electrophoresis; MSP, Methylation-Specific PCR.
