# Supplementary material for: The Novel Methylation Biomarker SCARA5 Sensitizes Cancer Cells to DNA Damage Chemotherapy Drugs in NSCLC
Source: Front Oncol. 2021 Jun 4;11:666589. doi: 10.3389/fonc.2021.666589 (PMC8213031; doi:10.3389/fonc.2021.666589)
Supplement: Supplementary file 1 [file Table_1.pdf]

Additional file 1：Table 1 List of Primers used in the present study

| PCR Primer | Sequence (5'-3') | Product size (bp) | Annealing temperature(℃) |
|---|---|---|---|
| FOXM1_F | GCTATCCCATCCATTTCTGTAGCC | | |
| FOXM1_R | GCAGACAGAAGCAATTCAAAGGGC | | |
| HSPA1A-F | TCCGGCGTCCGGAAGGACC | | |
| HSPA1A-R | TGCGGCCAATCAGGCGCTT | | |
| HSPA1B-F | CATCGACTTCTACACGTCCA | | |
| HSPA1B-R | CAAAGTCCTTGAGTCCCAAC | | |
| HSP5A-F | CGGTCTACTATGAAGCCCGT | | |
| HSP5A-R | CATCTGGGTTTATGCCACGG | | |
| SCARA5F | ACACCGTCAGCGACTGTGA | | |
| SCARA5R | GAAGATGCCCACAAGAATCAG | | |
| CHK1F | AGTGCCCTTTGTGGAAGACT | | |
| CHK1R | CTCCACTACAGTACTCCAGA | | |
| CDC25CF | GATGTCCCTAGAACTCCAGTG | | |
| CDC25CR | AGTTATCTCCCCACTGCTAAGA | | |
| CCNB1F | TCTGGATAATGGTGAATGGACA | | |
| CCNB1R | CGATGTGGCATACTTGTTCTTG | | |
| CDK1F | CGCAACAGGGAAGAACAG | | |
| CDK1R | CGAAAGCCAAGATAAGCAAC | | |
| GAPDHF | GGAGTCAACGGATTTGGT | | |
| GAPDHR | GTGATGGGATTTCCATTGAT | | |
| SCARA5m1 | TTTAAGGTTAAGGTCGATCGC | | |
| SCARA5m2 | TACTCTCGCCGCCAATCCG | | |
| SCARA5u1 | GTTTTAAGGTTAAGGTTGATTGT | 173bp | 60 |
| SCARA5u2 | ACTACTCTCACCACCAATCCA | | |
| | | 176bp | 58 |
