# Supplementary material for: The Novel Methylation Biomarker SCARA5 Sensitizes Cancer Cells to DNA Damage Chemotherapy Drugs in NSCLC
Source: Front Oncol. 2021 Jun 4;11:666589. doi: 10.3389/fonc.2021.666589 (PMC8213031; doi:10.3389/fonc.2021.666589)
Supplement: Supplementary file 2 [file Table_2.pdf]

Additional file 2: Table 2 The clinical information of lung cancer tissues.

No.	Age	Gender (1M2F)	Size (cm)	Score	T-stage	N-status	Distant Metastasis	Phase	Pathological type
1	68	1	2.9*1.5	3	2	1	0	IIB	LUSC
2	60	2	4.0*4.0	3	2	0	0	IIA	LUAD
3	56	2	Infiltrative	3	2	0	0	IIA	LUAD
4	44	2	3.6*3.3	3	2	1	0	IIB	LUAD
5	74	1	3.0*3.0	3	2	0	0	IIA	LUSC
6	57	1	7.2*6.0	2	4	2	0	IIIB	LUSC
7	55	1	2.0*1.8	2	2	0	0	IIA	LUAD
8	71	1	6.0*4.8	2	3	0	0	IIB	LUAD
9	55	1	6.0*5.0	1	3	2	0	IIIB	LUSC
10	61	2	3.8*4.1	1	2	2	0	IIIA	LUSC
11	52	1	3.0*2.5	1	2	2	0	IIIA	LUSC
12	32	1	3.2*3.0	1	2	0	0	IIIA	LUSC
13	49	2	3.0*3.5	1	2	0	0	IB	LUAD
14	63	1	3.0*2.0	0	2	0	0	IIA	LUSC
15	73	2	2.5*2.2	0	2	2	0	IIIA	LUAD
16	42	1	2.0*1.5	0	1	1	0	IIB	LUAD

Score:Expression of SCARA5

LUAD:Lung adenocarcinoma

LUSC:Lung squamous cell carcinoma
